# Supplementary material for: Molecular Aspects of Piperine in Signaling Pathways Associated with Inflammation in Head and Neck Cancer
Source: Int J Mol Sci. 2024 May 25;25(11):5762. doi: 10.3390/ijms25115762 (PMC11172343; doi:10.3390/ijms25115762)
Supplement: Supplementary file 1 [file ijms-25-05762-s001.zip › ijms-2959225-supplementary.pdf]

Supplementary Materials

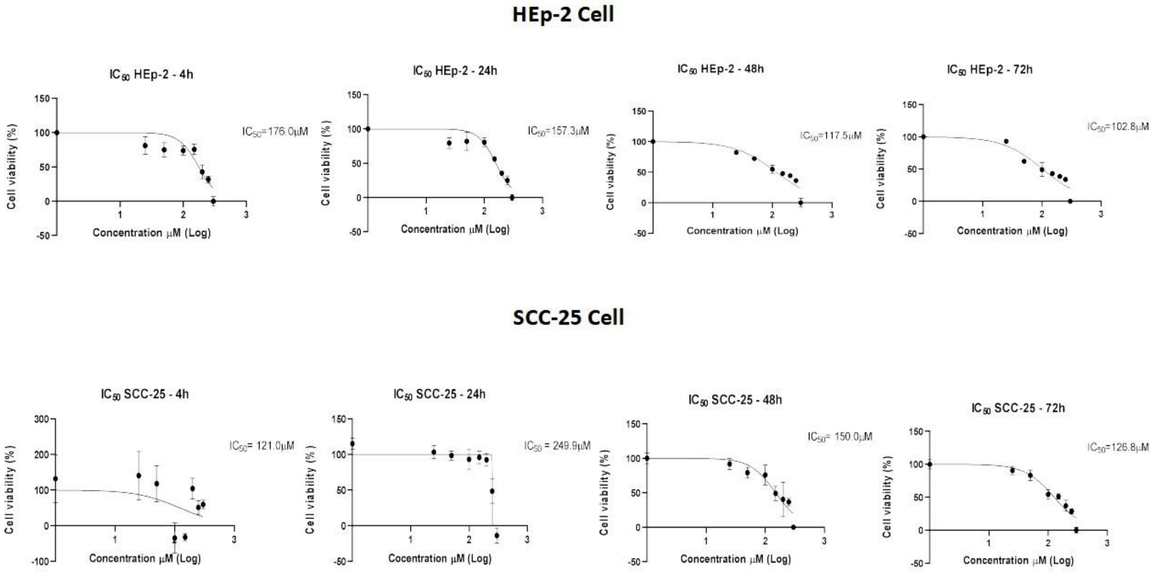

(**A**)

Concentration of compound that induces 50% cell death				
	4h	24h	48h	72h
HEp-2	176,0	157,3	117,5	102,8
SCC-25	121,0	249,9	150,0	126,8

(**B**)

Figure S1. Effect of piperine on cytotoxicity in HEp-2 and SCC-25 cells (**A**) at 4, 24, 48 and 72 hours, with IC_{50} showing the cytotoxicity index at 50% (Complementary material). Table with piperine inhibitory concentration values in HEp-2 and SCC-25 cells (**B**).
